# Strengthening health human resources and improving clinical outcomes through an integrated guideline and educational outreach in resource-poor settings: a cluster-randomized trial

**DOI:** 10.1186/1745-6215-11-118

**Published:** 2010-12-03

**Authors:** Michael J Schull, Hastings Banda, Damson Kathyola, Lara Fairall, Alexandra Martiniuk, Barry Burciul, Merrick Zwarenstein, Sumeet Sodhi, Sandy Thompson, Martias Joshua, Martha Mondiwa, Eric Bateman

**Affiliations:** 1 Dignitas International, 2 Adelaide Street West, Suite 200, Toronto, M5H 1L6, Canada; 2 Department of Medicine, University of Toronto, 200 Elizabeth Street, Toronto, M5G 2C4, Canada; 3 Department of Health Policy, Management and Evaluation, 155 College Street, Suite 425, Toronto, M5T 3M6, Canada; 4 Sunnybrook Health Sciences Center; 2075 Bayview Ave, Toronto, M4N 3M5 Canada; 5 Research for Equity and Community Health (REACH) Trust, POB 1597, Lilongwe, Malawi; 6 Ministry of Health Malawi, POB 3, Lilongwe, Malawi; 7 Knowledge Translation Unit, University of Cape Town Lung Institute, University of Cape Town, PO Box 34560, Groote Schuur 7937, South Africa; 8 George Institute for Global Health; University of Sydney, 341 George Street, Sydney, Australia; 9 Zomba Central Hospital, Kamuzu Highway, Zomba, Malawi; 10 Nurses and Midwives Council of Malawi, POB 30361, Lilongwe, Malawi

## Abstract

**Background:**

In low-income countries, only about a third of Human Immunodeficiency Virus/Acquired Immune Deficiency Syndrome (HIV/AIDS) patients eligible for anti-retroviral treatment currently receive it. Providing decentralized treatment close to where patients live is crucial to a faster scale up, however, a key obstacle is limited health system capacity due to a shortage of trained health-care workers and challenges of integrating HIV/AIDS care with other primary care services (e.g. tuberculosis, malaria, respiratory conditions). This study will test an adapted primary care health care worker training and guideline intervention, Practical Approach to Lung Health and HIV/AIDS Malawi (PALM PLUS), on staff retention and satisfaction, and quality of patient care.

**Methods/Design:**

A cluster-randomized trial design is being used to compare usual care with a standardized clinical guideline and training intervention, PALM PLUS. The intervention targets middle-cadre health care workers (nurses, clinical officers, medical assistants) in 30 rural primary care health centres in a single district in Malawi. PALM PLUS is an integrated, symptom-based and user-friendly guideline consistent with Malawian national treatment protocols. Training is standardized and based on an educational outreach approach. Trainers will be front-line peer healthcare workers trained to provide outreach training and support to their fellow front-line healthcare workers during focused (1-2 hours), intermittent, interactive sessions on-site in health centers. Primary outcomes are health care worker retention and satisfaction. Secondary outcomes are clinical outcomes measured at the health centre level for HIV/AIDS, tuberculosis, prevention-of-mother-to-child-transmission of HIV and other primary care conditions. Effect sizes and 95% confidence intervals for outcomes will be presented. Assessment of outcomes will occur at 1 year post- implementation.

**Discussion:**

The PALM PLUS trial aims to address a key problem: strengthening middle-cadre health care workers to support the broader scale up of HIV/AIDS services and their integration into primary care. The trial will test whether the PALM PLUS intervention improves staff satisfaction and retention, as well as the quality of patient care, when compared to usual practice.

**Trial Registration:**

Current controlled Trials: ISRCTN47805230

## Introduction

In 2008, UNAIDS estimated that 33 million people were living with HIV/AIDS [1]. Recent years have seen substantial improvements in access to antiretroviral treatment (ART). For example, in Malawi, a low-income African country, about 170,000 adults and children were alive and on ART in June 2009 [2]. The number of new ART initiations has increased year over year, yet worldwide, only about one-third of HIV patients who need ART are currently getting it [1]. Substantial efforts are now underway to further scale-up HIV/AIDS treatment in Malawi [2] and other resource-poor countries [1], and one key strategy is improving the scale and equity of access by decentralizing HIV/AIDS services to rural primary care centers [1,2].

A key obstacle to scaling up is health system capacity due to a shortage of trained workers in countries like Malawi. It has been estimated that Sub-Saharan Africa would require two times its current human resources to be added every year for the next 10 years to reach universal coverage for HIV/AIDS [3]. The goal of ensuring equitable access to quality health care is further hindered by the difficulty of retaining staff in rural areas of low- and middle-income countries; Malawi in particular has a HCW vacancy rate of 50% [4]. There is wide agreement that closing these gaps will require innovative strategies to optimize the use of existing human resources, and interventions to train and retain staff [4].

Attention to the quality of clinical care provided for HIV/AIDS and other primary care conditions while increasing access to HIV/AIDS services is also important. This is especially so since the workload inherent in a scale-up of access to HIV/AIDS services may have unintended negative consequences on existing primary care services in those same centers [3,5]. This study will test an adapted clinical guideline and training approach, Practical Approach to Lung Health and HIV/AIDS in Malawi (PALM PLUS), to strengthening the health care workforce in a primary care setting where HIV/AIDS services are being decentralized. PALM PLUS is designed to support mid-level HCWs, i.e. nurses and non-physician clinicians (in Malawi, medical assistants and clinical officers), with the overall goal of improving equitable access to quality HIV/AIDS care and primary care services. The primary objectives of the intervention are to improve mid-level HCW retention in rural health centres and improve their job satisfaction; secondary objectives are to improve patient outcomes. Our hypotheses are that the PALM PLUS intervention will be associated with: 1) improved staffing and staff satisfaction, and 2) improved patient outcomes.

## Methods/Design

### Study Design

A population-based unblinded stratified cluster randomized controlled trial design will be used to evaluate the PALM PLUS intervention. Health centers were selected as the unit of randomization in order to minimize contamination of program delivery as well as for logistical and pragmatic reasons. Outcomes of interest are at both the cluster and individual HCW's levels.

### Study Setting

The study is set in Zomba district, Malawi. *Inclusion criteria: *Clusters: All government, mission and institutionally funded health centers offering primary care services to the general public in the district (n = 30) are eligible (health centers are defined as those delivering primarily out-patient primary care, without elective surgeries or in-patient admissions of the general public, and ); individuals: all health professional staff eligible to provide clinical services within the randomized health centres will be included. *Exclusion criteria: *Cluster: none; individual HCWs: refusal to participate.

### Method of randomization

**Figure 1:**
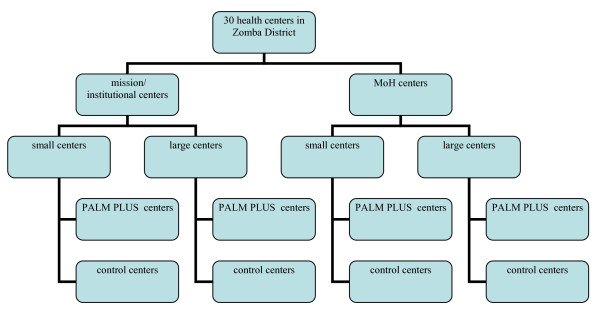
**Stratified randomization schematic, strata for randomization to intervention and control arm**.

Health centres will be randomized to the intervention (PALM PLUS) or the control arm (see Figure 1). Random allocations will be generated by the random selection option in the Minitab software program. Randomization will be done using two strata with two levels each. Prior to randomization, health centers will be stratified according to funding source (government funded, n = 15; vs. mission or institutionally funded, n = 15); and health centre size as defined by the number of health centre clinical staff postings defined as doctor, nurse, clinical officer or medical assistant working in the clinic at baseline (levels defined as < median number of HCW for all health centers or ≥median number). All health centers are under the supervision of the Ministry of Health's (MoH) District Health Office (DHO), submit performance data to the DHO, and all staff receive standard MoH training and supervision. This stratification is chosen because there may be subtle management, organizational, and motivation differences in government vs. mission/institutionally funded clinics, and larger staff numbers is a proxy for greater complexity of clinical programming and resources, as well as to ensure balance in terms of numbers of HCWs in both study arms. Randomization within strata will be restricted to ensure equal balance in intervention and control sites by health centre type.

### The Intervention

The intervention centers on the PALM PLUS guidelines and adapted training method, which are a Malawi-specific adaption of the proven PALSA PLUS guidelines implemented in South Africa [6]. PALM PLUS guidelines are symptom-based, integrated guidelines for HIV/AIDS, tuberculosis and common primary care conditions. They have been modified to comply with existing Malawian national disease and condition-specific guidelines, and through consultations with Ministry of Health personnel (at local and national levels), front-line Malawian nurses and clinical officers, and other stakeholders like the Medical Council of Malawi and the Nursing and Midwifery Council of Malawi. The reviewers are expected to check that the content is correct and appropriate and that the algorithms flow properly and reflect available drugs/resources in Malawian Health Centers. Guideline and algorithm development will follow a collaborative and iterative development process to ensure local relevance to front-line healthcare workers, to promote local ownership and to facilitate identification and resolution of any barriers to its implementation (e.g. inadequate access to diagnostic investigations for excluding TB). Key messages covering the content have been developed; these are short, summary information vectors, which provide a content framework for otherwise learner-directed teaching, and which are simple enough to be easily remembered during patient consultations.

Training follows the proven innovative and evidence-based model called educational outreach [7], a form of *point-of-care *training that provides case-based, on-site training to primary care providers in the clinical settings where they work. Trainers will be front-line peer healthcare workers (from government or mission/institutional health centers) who will be trained to provide outreach training and support to their fellow front-line healthcare workers during focused (1-2 hours), intermittent, interactive sessions. Two Master Trainers will be responsible for the initial facility-trainer training, which is conducted offsite during a one-week intensive course in which the facility trainers are equipped with the necessary content, and their training skills developed and evaluated using an iterative process of training and feedback.

Trainers will be expected to lead between 6 to 10 training sessions at each health centre, targeting all nursing and clinical staff working at the health centre, over a maximum period of 12 weeks, but they may conduct additional training sessions if they wish. Point-of-care guideline tools will be developed and distributed (e.g a thin (about 30 pages) sturdy laminated ring binder containing the entire guideline, colourful and easy to follow algorithms designed to be on the primary care provider's desktop during each patient encounter, as well desk blotters with key PALM PLUS messages.

### Control sites

No PALM PLUS materials or training will be provided to personnel working at control health centers. Routine ongoing health centre supervision by District Health Office (DHO) personnel and off-site DHO training courses will continue in all sites.

### Outcome measures

Our primary outcomes will be measured at the cluster (health centre) and individual (staff) levels and relate to clinical staffing (clinical officers, medical assistants, nurses): 1) retention (staff continuing to work in same health centre), 2) total staffing (total of retention/recruitment/loss), and 3) staff satisfaction at the cluster and individual level. Secondary outcomes are clinical outcomes targeted by the PALM PLUS guideline measured at the health centre level: 1) total patient visits, 2) use of cotrimoxazole prophylaxis among HIV+ pregnant women, 3) HIV testing among pregnant women, 4) ART provision among HIV+ pregnant women, 5) Non-ART prevention-of-mother-to-child-transmission treatment provided to HIV+ pregnant women, 6) new adult ART initiations, 7) new adult TB treatment initiations, 8) new adult TB treatment initiations among ART patients, 9) new HIV diagnosis among TB patients, 10) new ART initiations among TB patients, 11) administration of malaria treatments. We will also evaluate competing pediatric primary health priorities as potential unintended consequences (since PALM PLUS targets only the care of adults): 1) total and measles-specific immunizations given 0-5yrs, 2) new ARV initiations among children, 3) HIV tests among children.

### Ascertainment of outcomes

Outcomes will be ascertained at baseline and during monthly visits to all health centers; main comparison will be at 12 months vs. baseline;. The clinical manager will be interviewed each visit to determine ongoing employment, and dates of new arrivals and departures of clinical staff. A validated staff satisfaction survey [8] will be administered to clinical staff (at baseline, 2-4 months [for immediate staff satisfaction/retention evaluation], and 10-12 months [late outcomes], ensuring that data is collected for approximately the same calendar months at study and control sites. Patient outcomes will be determined by monthly review of health centre registers and/or patient treatment cards.

#### Analysis plan

In cluster randomized controlled trials with relatively few clusters, it is recommended to assess the effectiveness of randomization as a first step. Therefore, we will first examine the balance of baseline characteristics of the health centre clusters and the individual patients across intervention groups. Analyses will be done at both cluster (health centre) and individual (health care worker) levels, adjusted for clustering. At the health centre level (cluster level), we will use multivariable linear regression to compare mean staff satisfaction, staff retention and total staffing, adjusting for potential confounders. At the health worker level (individual level), multivariable regression adjusted for clustering by health centre will be used to compare HCW satisfaction scores. For this analysis, we will only include those HCWs who complete a survey at baseline and the appropriate outcome time point and are working in the same health center. Cox regression modeling will be used to estimate the survival probabilities of health care workers (proportion of days employed at health centre over two years of follow-up) to compare 'survival' (sustained days of work at same health centre) across intervention groups, adjusting for potential confounders. Health centre level analyses will use multivariable linear regression to compare secondary outcomes such treatment initiation (ARV, cotrimoxazole, TB, malaria etc.). Treatment initiation will be calculated as the number of patients placed on "X" treatment over the denominator of "Y" health centre referral population. To adjust for cluster randomization, we will use generalized estimating equation binomial regression models with log link and exchangeable correlation structures to estimate the relative risks (RR) and 95% confidence intervals for 'survival' rates (proportion of days employed at health centre over two years of follow-up) between the intervention group (receive PALM PLUS) with the control group as a reference. SAS version 9.2 (SAS Institute Inc, 2008) will be used for all analyses. All analyses will be conducted using the intention to treat principle.

#### Potential confounders at the cluster level

Potential confounders at the cluster level: phase of ART services (stand-alone ART provision vs. any other phase) measured at baseline and number of PALM PLUS training sessions at health centre (< 10 (i.e. less than maximum expected number of trainings) vs ≥10 training sessions, determined at the end of the intervention period. Number of training sessions will not be included in our primary analysis, but only in a secondary analysis as it is a post-randomization variable. *Potential confounders at the individual level: *Age, gender, staff cadre (clinical officer, nurse, medical assistant), staff grade (professional vs. technical), years working in profession, born in Zomba region (yes/no), number of PALM PLUS training sessions attended, and exposure to other MoH trainings (number of days of training); the latter two confounders will be determined at the end of the intervention period.

#### Sample size Calculations

At a two-sided alpha of 0.05 and holding power at 80%, using an estimated value for the intracluster correlation coefficient (ICC) of 0.15, and p1 of 0.90 and p2 of 0.65, an inflation factor of 0.15 and an average cluster size of 5 workers then 67 workers will be required per study arm (the sample size unadjusted for clustering is 42 workers). Inflating this by 10% to ensure a sufficient number of staff have completed multiple longitudinal satisfaction surveys within the same health center, brings the required sample to 74 workers which is equivalent to needing 15 clusters (health centers) per study arm. Rationale for ICC: The ICC is almost always unknown prior to conducting a study. In this instance we have chosen an estimate for the ICC to be 0.15. The rationale for the selection of this ICC value is that previous studies have shown higher ICC values for process variables (e.g., health worker days) compared to outcome variables in the primary care setting [9] and in a previous study which summarized the median ICC from several studies using process variables, the median was found to be 0.2 [10].

### Ethical considerations

The study has been approved by the National Health Sciences Research Committee, Malawi's national research ethics board. The study subjects in this study are health centre personnel. Health centre staff receive routine training and supervision from the Ministry of Health (MoH) based on curricula developed and approved by the MoH. The PALM training curricula and materials will be reviewed by the MoH for training purposes. Control and intervention sites will continue to receive routine MoH approved training and supervision, while only intervention sites will receive PALM training. Individual consent from health centre staff is not required because undergoing training is a routine expectation of health centre staff, and the PALM PLUS guidelines will be fully consistent with existing national guidelines, and will be reviewed by key MoH staff. We will seek consent from health centre staff to ask them to complete questionnaires on job satisfaction, and/or participate in a focus group or interview examining job satisfaction. To ensure confidentiality of the staff responses, only unique numeric identifiers will be used for HCWs, and at no time will their identity or their responses be disclosed. The list of codes and corresponding staff names will be kept separately in a secure location accessible only to the study team. Any paper documents and audio recordings of focus groups will be stored in a lockable cabinet in a locked room. All electronic documents will be password protected. Access to the data will be limited to the investigation team and will be used solely for the study purposes. The conclusions of the study will be used to inform program improvements and will not compromise the privacy, confidentiality or well-being of the human subjects whose data will be used. All data will be destroyed five years after the completion of the study.

## Discussion

The burgeoning load of ART patients at rural health centres in Malawi has placed an increasing burden on health care staff in those centers, in an area where there is a 50% vacancy rate among clinical posts [4]. This reflects the situation across sub-Saharan Africa, which suffers from the lowest HCW-to-population ratio in the world [4]. This burden is added to the existing demand for non-HIV primary care. Current models of HCW resource needs often look at HIV/AIDS care in isolation [3], without factoring in the need to provide other care. The recognition of the risks of a vertical approach to health services is not new [11], however the push for more rapid scale-up and decentralisation of HIV/AIDS services, their current lack of integration with other primary care, and the potential for additional disease-specific vertical programs in Malawi [12], makes integration at the primary care level an even more pressing priority.

This study will respond to these facts by developing and evaluating a targeted intervention to optimize the clinical effectiveness of, and improve the satisfaction and retention of, healthcare workers in rural health centers. This will be accomplished through the collaborative development, implementation and evaluation of an integrated clinical guideline and training intervention that will facilitate the integration of adult HIV/AIDS and TB care with primary care. Current national disease-specific guidelines in Malawi, and many other countries, are not integrated: each is developed and implemented largely in isolation, and some discrepancies between different disease-specific guidelines exist which can confuse clinicians. Recommendations in national guidelines may be impossible to implement in small health centers due to lack of access to recommended tests or treatments at the primary health centre level. Traditional in-service training is often also disease-specific. In some conditions such as TB and HIV/AIDS, clinical integration has begun to occur, but more comprehensive adult integrated guidelines and tools to assist the nurse or clinician at the bedside have not yet been developed. Disease-specific guidelines and training may be quite appropriate at specialized clinics in larger centers, but this model provides limited support to front-line nurses and clinicians in primary care health centers.

Our study is focused on the implementation of PALM PLUS, a single set of adapted, integrated symptom- and sign-based primary care guidelines for adults, combined with an innovative training program for health care workers. We will test whether PALM PLUS is associated with improved staff retention and satisfaction, and improved patient care, when compared with usual practice. PALM PLUS is not designed to replace national disease-specific guidelines, but rather to assist nurses and clinicians at health centers to use existing national guidelines and protocols more effectively in their day-to-day primary care patient encounters. The foundation for this work rests in the PALSA-PLUS guidelines, which were developed by researchers at the Knowledge Translation Unit of the University of Cape Town Lung Institute in an evidence-based, highly collaborative and consultative process [6]. In rigorous studies, the implementation of PALSA-PLUS with nurses in health centers in South Africa has been shown to improve the case detection of tuberculosis by 68%, the treatment of asthma by an 80% increase in appropriate provision of inhaled steroids and a 120% increase in appropriate referral of severe asthma, and a 110% increase in HIV voluntary counseling and testing among TB patients [7]. The PALSA guideline and training also effectively identified patients requiring screening for pulmonary tuberculosis in primary care settings [13]. More recent research in South Africa has demonstrated that PALSA PLUS led to improved outcomes for HIV patients as well: a cluster randomized trial found improved provision of cotrimoxazole prophylaxis against pneumocystis pneumonia in HIV+ patients, and improved TB case detection [Zwarenstein M., personal communication, article in press]. Just as important, studies of PALSA-PLUS training have shown high nurse uptake of ART and primary care training, enhanced nurses' experience of support from their managers, better emotional and operational support, and increased confidence and better horizontal integration of HIV/AIDS care with routine primary health care [14,15]. These are all factors which may affect HCW satisfaction, recruitment and retention in countries such as Malawi [4,14-16], a key factor slowing the scale-up of access to high quality HIV/AIDS care in many countries.

## Competing interests

The authors declare that they have no competing interests.

## Authors' contributions

MJS, SS, ST and BB conceived of the study. MJS, SS and BB led the drafting of the protocol and grant applications. LF, MZ, EB, MZ, MJ, AM and HB participated in study design. DK and MM provided policy support and commented on drafts. All authors read and approved the final manuscript.
